# Nasal Polypus

**Published:** 1851-01

**Authors:** 


### ARTICLE XVII.

### *Nasal Polypus.*

Mr. Muggridge, in a communication to the Lancet, says: I was requested to see a female, S. R., aged twenty-two years, who had a nasal polypus, which was daily increasing in size, although it had been operated on twice, at intervals of eight months, at a public hospital. The first time my patient was admitted an in-patient for a month, as she informed me, to prepare her for manual interference; and from her statement it appears, that at each operation it came away in little bits—piecemeal. It is now eight months since she was last operated on: the polypus began to get very troublesome and large, filling up the left nostril, and producing headache, difficulty of breathing, and, as she described it, a great snuffling during sleep, thereby preventing any one sleeping in the same room. On introducing the probe, I found it extended to the upper part of the superior turbinated bone. I extracted it with the common dissecting forceps. I got a pretty firm hold, by introducing them as high as possible, and, by a gentle twist, and a sudden jerk, (as recommended by the late Sir Astley Cooper,) it came away entire. It was of an hour-glass shape; the lowest portion which could be seen is about four inches in circumference, attached by a peduncle to the upper, which is larger, and of a more irregular form; on each portion of it were small red vessels. My opinion is, that the lower portion only, below the peduncle, had been extracted before, which caused it to form again, while the upper portion adhered to its attachment. There was very little hemorrhage. Before leaving my patient, I stuffed the nostril with lint dipped in liquor aluminis. In the evening I again saw her, and found the lint had come out: she was much relieved, no headache, and she could breathe freely and easily through the nostril, which, on being looked into, appeared as free from disease as the other.

